# Effect of Hypoxia on the Lethal Mortality Time of Adult *Sitophilus oryzae* L.

**DOI:** 10.3390/insects12100952

**Published:** 2021-10-19

**Authors:** Pragya Kandel, Michael E. Scharf, Linda J. Mason, Dieudonne Baributsa

**Affiliations:** Department of Entomology, Purdue University, W. Lafayette, IN 47907, USA; pkandel@purdue.edu (P.K.); mscharf@purdue.edu (M.E.S.); lmason@purdue.edu (L.J.M.)

**Keywords:** grain storage, insect pests, controlled atmosphere, biological mortality

## Abstract

**Simple Summary:**

The rice weevil is a major pest of stored grains that leads to losses resulting in food and income insecurity among farmers. Pesticides, often used by farmers to control insect pests of stored products, are becoming unattractive due to health risks to applicators, consumers, and the environment. Hermetic (airtight) storage methods have been used as alternatives to pesticides. Understanding when insects die during hermetic storage is vital, in order to improve pest management. We conducted experiments to assess the time required to attain mortality of adult rice weevils when the oxygen levels reached below 5% in airtight containers. Results revealed that it required 69.7, 187.8, and 386.6 h to kill 50% of adult rice weevils exposed to 1%, 3%, and 5% oxygen levels, respectively. No adult emerged from infested grains following exposure to 1 and 3% oxygen levels, but some did at 5% oxygen level. Based on these results, we recommend that grain be kept in hermetic airtight conditions for at least 39 days to achieve adult rice weevil mortality and minimize grain reinfestation.

**Abstract:**

*Sitophilus oryzae* is one of the most destructive pests of stored grains. It leads to significant quantitative and qualitative losses, resulting in food and income insecurity among farmers. Chemical pesticides are the most common methods used by farmers and other grain value chain actors to manage this pest. However, pesticides are increasingly becoming unattractive for pest control due to health hazards posed to applicators, consumers, the environment, and insect resistance. Modified atmospheres have the potential to manage stored insect pests as an alternative to pesticides. There is limited understanding of when insect pests die when grain is stored in airtight containers. This experiment was conducted to assess the time required to reach mortality of adult *S. oryzae* when exposed to 1, 3, and 5% oxygen levels. Results revealed that the LT50 for 1, 3, and 5% of oxygen were reached after 69.7 h, 187.8 h, and 386.6 h of exposure, respectively. No adult emergence was observed on infested grains following exposure to 1 and 3% oxygen levels. This result provides vital rationale for storing grain in hermetic storage conditions for at least 39 days to achieve adult *S. oryzae* mortality and minimize grain reinfestation.

## 1. Introduction

*Sitophilus oryzae* (Linneaus) is a destructive and widespread primary pest of stored cereals and legumes. It feeds on a wide range of grains and processed foodstuffs including rice, wheat, barley, corn, and sorghum [1,2]. The damage caused by *S. oryzae* is mostly due to the feeding activities of adults and grubs [3]. Thus, the damage caused by *S. oryzae* results in losses of quantity and quality, leading to a reduction in market value [4]. Most farmers use a variety of strategies to manage *S. oryzae* during grain storage, including pesticides such as phosphine fumigants, organophosphorus, and pyrethroid insecticides [5,6]. The use of chemical pesticides often comes with many challenges including ineffectiveness due to insect resistance or misapplication, health hazards to applicators and consumers, and environmental contamination [7,8]. The widely used fumigant methyl bromide was phased out due to its high toxicity and depletion of the ozone layer [9].

Since the early 1980s, modified atmospheres have been explored as possible alternative protection methods to traditional fumigants against stored-product insect pests [10,11]. Hermetic storage is one of these modified atmospheres that relies on natural processes such as respiration by insects and other biological activities to deplete oxygen, leading to insect mortality [1,12,13]. Experiments conducted to understand the lethality of insects under controlled environments have shown that gases such as oxygen (O2), carbon dioxide (CO_2_), and nitrogen (N_2_), either alone or in various combinations, can partially or fully control insect pests during storage [14,15,16,17]. Studies have shown that oxygen levels below 5% can suppress *S. oryzae* activities and adult emergence for most insect pests of stored products [11,18,19]. Lowering the oxygen level to 2% was shown to cause complete mortality in 3 days for eggs, 7 days for young larvae, 10 days for old larvae and pupae, and 15 days for adults [19,20]. These studies show the effectiveness of modified atmosphere as an alternative to pesticides.

Field studies have shown that hermetic storage methods are effective at maintaining grain quality, though on a few occasions there are reports of insect survival after several months of grain storage [21]. Insects exposed to a 4% oxygen level for 15 days can still survive and develop progeny [20]. Acoustic studies conducted under hypoxic environments have provided some insight into insect behavior under low oxygen conditions. Exposure of *S. oryzae* to hypoxia below 5%, caused their “acoustical death” within five days [18], which means simply that their rate of sound bursts had fallen below the threshold for a low likelihood of infestation [22]. However, there is little understanding of when insects are biologically dead under hypoxic conditions, when grain is stored in airtight containers. This experiment was conducted to assess (i) the effect of oxygen levels below 5% on the biological mortality of rice weevils, and (ii) progeny development following exposure to oxygen levels below 5%.

## 2. Materials and Methods

This study was conducted in the department of entomology at Purdue University in Indiana. The experiments were implemented in the Purdue Improved Crop Storage (PICS) laboratory from Spring 2020 to Spring 2021.

### 2.1. Insect Rearing

Insects used in this trial were reared in a CONVIRON insect growth chamber (Model CMP4030; CONVIRON., MB, Canada). The wheat variety AG 1189 (Alumni Seed Co., Romney, IN, USA), stored at −18 ± 1 °C for disinfestation, was used in the experiment. Before the colony was set up, the wheat was thawed for 24 h at 20 ± 1 °C. *S. oryzae* were taken out of an existing colony using a vacuum aspirator and then transferred to one-liter jars containing wheat. Insects were allowed to breed for 48 h and then removed using a vacuum aspirator. After removing beetle adults, the grain was kept in a growth chamber at 25 ± 1 °C and 40 ± 5% relative humidity (RH) until adult emergence. Newly emerged *S. oryzae* adults (3–5 days) were used for the experiment. A no. 10 sieve was used to remove adults from the grain. Ten adults each were transferred into 30 mL containers/vials (Wheaton Glass Sample bottle, CP Lab Safety, Novato, CA, USA).

### 2.2. Hypoxia Treatment

The treatments consisted of three oxygen levels: 1, 3, and 5%. The level of hypoxia in the chamber was created by flushing the air out of the chambers and replacing it with nitrogen gas (Airgas, Kokomo, IN, USA) from a gas cylinder until the target oxygen concentration of the chamber was attained. An Oxysense 5250i oxygen reader device (Industrial Physics, Devens, MA, USA) was used to measure the concentration of oxygen inside the chamber, through fluorescent yellow Oxydots attached to the inner surfaces of the chamber. Oxygen content was maintained within ±1.00% of the target oxygen level. For maintaining the desired oxygen level once samples were removed, nitrogen was pumped back in to obtain the desired oxygen level within five minutes.

### 2.3. Experimental Setup and Design

The three oxygen levels were each maintained at six different periods: 48, 72, 96, 120, 144, and 168 h for 1%; 120, 144, 168, 192, 216, and 240 h for 3%; 168, 192, 216, 240, 264, and 288 h for 5%. The exposure periods for each oxygen level were set based on preliminary experiments that assessed when insect mortality began for *S. oryzae* under each oxygen level. Each treatment and the control were replicated thrice.

The experiment was conducted at a room temperature of 20 ± 1 °C. Clear polycarbonate vacuum chambers (41.9 cm × 34.5 cm × 38 cm) with 35 L capacity (Bel-Art Industries, Wayne, NJ, USA) were used to expose the adult *S. oryzae* to different levels of hypoxia. Ten recently emerged adult *S. oryzae* were put in a 30 mL container that contained 10 ± 0.5 g of wheat. To ensure gas exchange occurred when placed in the hypoxia chambers, each container was covered with a perforated lid with small holes. Five containers for each treatment group were placed in a vacuum chamber, and this was replicated three times (three vacuum chambers, total n = 15). The control was kept under a normoxic (20–21%) environment and replicated only once for each treatment. Each replication of the experiment consisted of the one oxygen level and all the time periods, given the limited number of vacuum chambers. During the experiment, each chamber had two treatments (the same oxygen level for two different times) inside it. For example, a vacuum chamber at 1% oxygen level held five containers for 48 h and the other five containers were kept in for 72 h. A second vacuum chamber at 1% held five containers for 96 h and the other five containers were held for 120 h. A third vacuum chamber at 1% held five containers for 144 h and the other five containers were held for 168 h. Each chamber with these treatments was replicated three times (a total of nine chambers). The experiment was repeated twice for increased accuracy and minimized error.

### 2.4. Data Collection

#### 2.4.1. Adult Mortality

After the treatments were taken out from the hypoxia chambers, each container was then emptied onto white paper to assess whether the *S. oryzae* adults were alive or dead. A *S. oryzae* adult that started moving immediately once exposed to normoxia was recorded as alive and kept in a separate container. Immobile adults were touched using forceps. If they showed any movement following the touching they were recorded as alive. Lastly, *S. oryzae* adults that did not respond were kept in the growth chamber further at 25 ± 1 °C and 40 ± 5% RH for 24 h, when they were assessed again to determine whether or not they were alive. This was done to ensure that insects were dead and not in hypoxic stress.

#### 2.4.2. Adult Emergence

To assess progeny from insects exposed to different treatments, grains in the five 30 mL containers were combined in 450 mL containers (Wheaton Glass Sample bottle, CP Lab Safety, Novato, CA, USA). Hence, there were three containers for each treatment plus the control. The four, 450 mL containers for each treatment were incubated in Caron insect growth chambers (Model 6025-1, 115 VAC, Caron Growth chambers, OH, USA) at 25 ± 1 °C and 40 ± 5% RH for 45 days. After 45 days, adult emergence was recorded for each treatment (three replications along with control).

#### 2.4.3. Temperature and RH

A USB data logger (Lascar, Erie, PA, USA) was kept inside each of the nine vacuum chambers to monitor temperature and RH. Two data loggers were kept outside of the chambers to monitor the room’s ambient atmosphere. The temperature and RH both inside and outside of the chamber were recorded every 30 min for the duration of each treatment.

### 2.5. Statistical Analysis

The mortality from each treatment replicate (container) was assessed and recorded. As each container had just 10 insects, all *S. oryzae* adults in the five containers were added to make the sample size normal i.e., 50 adults. A generalized linear model (Pearson’s test and likelihood ratio) was conducted to check the fitness of the model [23]. After that, the R package “Ecotox” was used to calculate the lethal time [24]. For adult emergence, the count was transformed using a square root transformation to better fit the data into a linear model. Next, a Tukey’s test was conducted to compare the means of adult emergences for each oxygen level. The means were separated using Bonferroni adjustments at *p* < 0.05.

Temperature and RH data from all of the hypoxia chambers were downloaded from the data loggers as Excel files. The daily averages were calculated by averaging the data of every 30 min taken from the data loggers. The daily averages for the longest exposure times were then plotted into Excel for 1, 3, 5%, and the control for the first seven days to match the duration of the 1% oxygen treatment. A Tukey’s test was conducted to compare the means of RH within the treatments and control.

## 3. Results

### 3.1. Lethal Time

The average mortality of *S. oryzae* across different treatments (exposure time within each oxygen level) is presented in Table 1. There were significant differences in *S. oryzae* adult mortality between the treatments and controls within each oxygen level. At the 1% oxygen level, the maximum average adult mortality of 100% was observed within 120 h of exposure. At 3%, the average adult mortality of 100% was attained within 264 h of exposure. The average adult mortality was only 21% at 5% of oxygen after exposing the insect for 288 h.

The analysis of the lethal time of *S. oryzae* adults exposed to 1, 3, and 5% of oxygen is shown in Table 2. The LT50 and LT99 of *S. oryazae* adults exposed to hypoxia at 1% were up to 3 days and 5 days, respectively, and were significantly different (*p* < 0.05). The data shows that 100% of weevil mortality was achieved within 5 days of the exposure to hypoxia. The LT50 and LT99 for 3% of hypoxia were up to 8 days and 11 days, respectively, and were significantly different (*p* < 0.05). At 5% of hypoxia, the LT50 for adult rice weevils was up to 20 days and the LT99 was up to about 39 days, and these were significantly different (*p* < 0.05).

**Figure 1:**
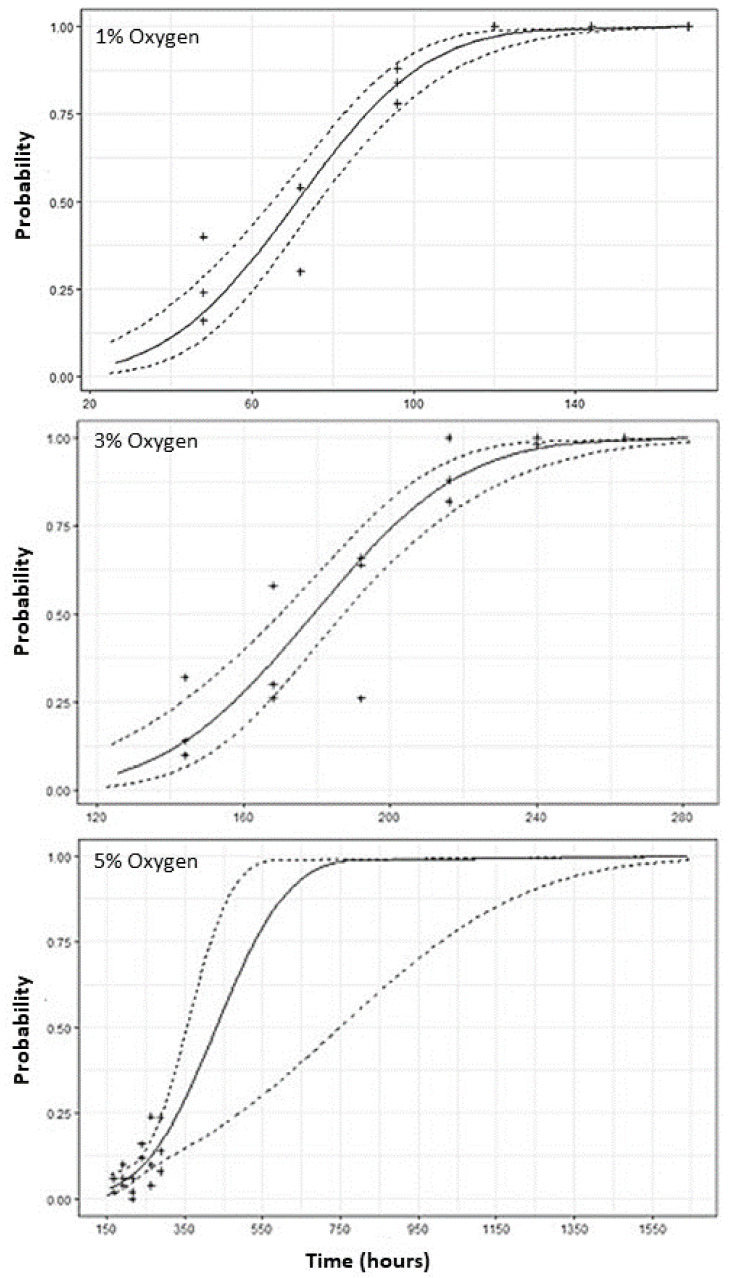
The probability of the mortality of *S. oryzae* with respect to time (hours) at 1, 3, and 5% oxygen levels. The dotted line represents the upper and lower level of 95% confidence interval of regression analysis.

The probabilities of adult mortality when exposed to 1, 3, and 5% oxygen levels for different times are shown in Figure 1. Although the slope is low for these levels of hypoxia in Table 2, the graph strongly supports our assumption of higher adult mortality with increased exposure to low oxygen levels. Figure 1 shows that most of the data points at 1% and 3% are within the confidence levels and well distributed throughout the graph. In contrast, at the 5% oxygen level, most of the data points are centered around the starting point in the curve, as 100% mortality was not attained during the experiment. This skewed distribution of mortality values is a plausible reason for the widened confidence level limits that were obtained in the LT analysis.

### 3.2. Adult Emergence

**Figure 2:**
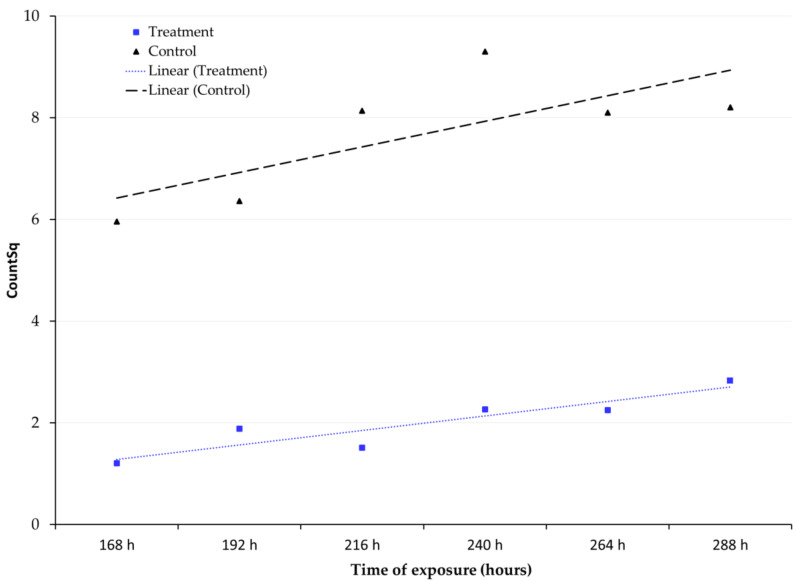
Insect emergence from the treated grain after exposure to 5% oxygen at 25 ± 1 °C and 40 ± 5% RH for forty-five days. “Countsq” refers to the square rooted value of adult insects that emerged after exposure to normoxic conditions following hypoxia treatment.

There was no adult emergence on grains exposed to 1% and 3% oxygen levels. At 5%, however, there were some emergences from the grains that were used in the treatment. Figure 2 shows the difference in the square root of the number of adult emergences in treatments and controls, along with the trend lines. Adult emergence at the 5% oxygen level was much lower when compared to control. The means ± standard deviation of adult emergence for the different exposure time periods at 5% were 1.2 ± 0.0, 1.9 ± 0.4, 1.5 ± 0.8, 2.3 ± 1.2, 2.2 ± 1.0, and 2.8 ± 0.9 adults for 168, 192, 216, 240, 264, and 288 h, respectively. The trend line shows a slight increase in adult emergence with longer exposure times, though it was minimal. A Tukey’s test comparing means at *p* < 0.05 showed no significant difference in adult emergence among the various time periods at 5%.

### 3.3. Temperature and RH

**Figure 3:**
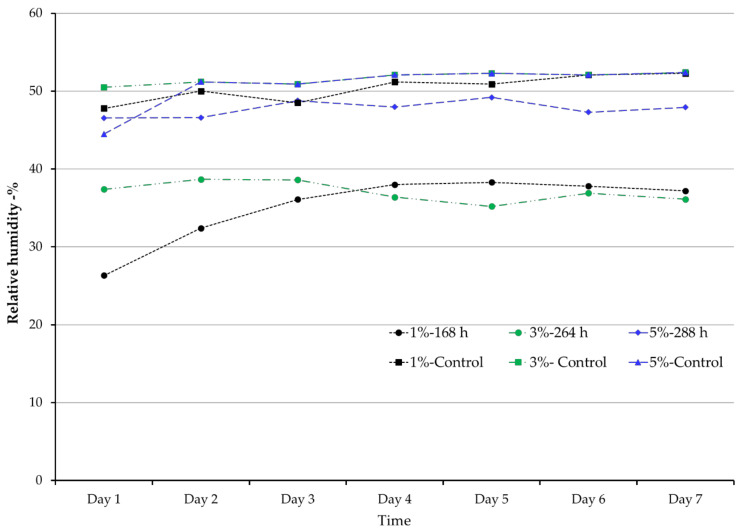
Average relative humidity at different oxygen levels within the first seven days of the maximum duration for each treatment and its control.

The temperature was 20 ± 0.5 °C throughout the experiment regardless of oxygen level. The average RH ± standard deviation for the first 7 days of each oxygen level were 35.2 ± 4.4% and 50.4 ± 1.7% for 1% and its control; 37.0 ± 1.3% and 51.6 ± 0.8% for 3% and its control; 47.8 ± 1.0% and 50.8 ± 2.8% for 5% and its control. The average relative humidity was significantly different between treatments and controls at each oxygen level (*p* < 0.05). However, no significant differences were observed among treatments within each hypoxia level. Figure 3 only shows the RH over seven days of treatments that were kept in the oxygen chambers for the maximum duration of each oxygen level. Relative humidity significantly decreased in treatments exposed to 1% and 3% oxygen when compared to those exposed to 5%.

## 4. Discussion

The mortality of *S. oryzae* followed the trend of the cessation of insect acoustic activity which was previously observed at the 1% oxygen level [18]. The low adult mortality in the first two days of the experiment is corroborated by the high insect sound burst rate observed in a previous study [18]. By the fourth day, *S. oryzae* mortality at the 1% oxygen level increased to 90% and is in line with the result of their acoustic activities with a threshold level below 0.002 bursts/sec [22]. This finding is substantiated by previous studies that showed that an oxygen level of 1% resulted in rapid death of the insects [12,25]. Following exposure to hypoxia treatments, no adults of *S. oryzae* recovered after being kept in a normoxic environment for 24 h. This suggests that the immobility of *S. oryzae* was an indicator of the actual insect mortality.

At the 3% oxygen level, insect mortality observed in this study did not show a similar trend of the cessation of insect acoustic activity as previously reported [18]. This study suggested a gradual decline in the rate of bursts in the first three days of exposure to hypoxia, and reaching a threshold level below 0.002 bursts/sec by the fourth day [22]. The mortality of *S. oryzae* reached 15.67% in six days and was not significantly different from the control. It took ten days to reach above 90% *S. oryzae* adult mortality. Movement of *S. oryzae* was not detected from the visual observation after the fourth day of exposure, but the weevils were still alive. This further signifies the need to understand the timeframe of hypoxia exposure necessary for effective elimination of insects. If the insects are not dead and are just immobile, it is likely they would feed and breed again as soon as they are exposed to normoxic conditions [20].

At the 5% oxygen level, the mortality of insects was significantly lower. Even though 100% mortality of *S. oryzae* was not achieved at 5% of oxygen, the mean timeframe for the cessation of acoustic activities is between days four and five [18]. Our results show that *S. oryzae* needs to be exposed to a 5% oxygen level for 39 days to achieve LT99 adult mortality. Otherwise, live insects may be observed after several weeks of storage if airtight conditions are breached. This finding provides good insight in terms of controlling *S. oryzae* under hermetic storage conditions. Oxygen levels of 5% or below are attainable in hermetic bags such as the Purdue Improved Crop Storage (PICS) bags [26]. Oxygen levels of around 5% have proven to be effective in eliminating insect infestations in hermetic bags if maintained for several months [1,4,26]. Under these circumstances, hermetic storage can produce similar results to those obtained under controlled atmospheres [18].

Our result on adult emergence substantiates previous findings which showed that oxygen levels below 5% can suppress the adult emergence of pests in stored products [20,27]. In our study, no adults emerged within 45 days post-treatment of grains exposed to 1% and 3% oxygen levels. The 5% oxygen level had some adults emerge, but it was significantly lower compared to those observed in grain exposed to normal oxygen levels. Hypoxia slows down the overall population growth of insect pests by reducing their mating, oviposition, progeny development, and longevity [20,27]. Further work needs to be done on understanding the factors that inhibit adult emergence in prolonged exposure to hypoxia.

The use of modified storage is often associated with low RH [11]. In contrast to the results of a previous study [20], we found that RH inside the chamber was affected by the level of hypoxia. The average RH was lowest at a 1% oxygen level, followed by 3% and 5%. Even though there was a slight increase in RH over time, we found that the addition of nitrogen sharply decreased the RH. Maintaining the oxygen level at 1% required frequent pumping of nitrogen gas compared to 3% and 5%. A combination of *S. oryzae* activity with the regular addition of nitrogen gas, such as low metabolic water production, might be the reason for the significant gap between treatments and controls. Further research is needed to assess the effect of low hypoxia levels on RH, and its potential impact on insect mortality and grain moisture content.

## 5. Conclusions

Exposing *S. oryzae* to an oxygen level of 1% and 3% for 5 and 11 days, respectively, was effective in causing 100% mortality. On the other hand, 5% hypoxia did not achieve complete mortality of adults, but prevented insect damage and reduced adult emergence following the treatment. The present study provides a vital rationale for storing grain in hermetic storage technologies for at least 39 days to achieve 99% of adult *S. oryzae* mortality and to minimize grain reinfestation.

## Figures and Tables

**Table 1:** Average mortality of *S. oryzae* adults exposed to 1, 3 and 5% oxygen levels at different time periods. Each treatment (combination of oxygen level and time period) had 5 samples replicated 3 times (n = 15), except for the control which had only 5 samples (n = 5). The whole set of experiments was repeated twice leading to total samples, n = 30 for each treatment and n = 10 for control. Means within rows with the same letter are not significantly different at *p* < 0.05.

1%	Time	48 h	72 h	96 h	120 h	144 h	168 h	Control
	Mortality (%)	20.33a	46.67b	89.33c	100c	100c	100c	2.83a
3%	Time	144 h	168 h	192 h	216 h	240 h	264 h	Control
	Mortality (%)	15.67ab	30.33bc	42.67c	75.33d	97.67e	100e	1.17a
5%	Time	168 h	192 h	216 h	240 h	264 h	288 h	Control
	Mortality (%)	4.33a	7.67a	8.67ab	14.67bc	16.33bc	21.00c	1.00c

**Table 2:** Time mortality regressions for *S. oryzae* adults exposed to different oxygen levels.

% Oxygen	LT_Value ^a^	N	Mortality (h)	LCL ^b^	UCL ^c^	χ^2 d^	DF ^e^	Slope
1%	25	1600	54.9	49.4	59.3	102.8	34	0.046
	50	1600	69.7	65.9	73.3			
	99	1600	120.6	112.9	131.3			
3%	25	1600	164.2	155.3	171.3	169.5	34	0.029
	50	1600	187.8	181.6	193.7			
	99	1600	269.0	256.1	287.2			
5%	25	1600	298.3	279.3	333.2	49.7	34	0.0076
	50	1600	386.6	352.2	474.8			
	99	1600	691.4	575.8	925.6			

^a^ Lethal time value; ^b^ Lower confidence level; ^c^ Upper confidence level; ^d^ Chi square value; ^e^ Degree of freedom.

## Data Availability

Raw data is not publicly available.
